# Starvation signals in yeast are integrated to coordinate metabolic reprogramming and stress response to ensure longevity

**DOI:** 10.1007/s00294-017-0697-4

**Published:** 2017-04-25

**Authors:** Nianshu Zhang, Lu Cao

**Affiliations:** 1 Cambridge Systems Biology Centre and Department of Biochemistry, University of Cambridge, Sanger Building, 80 Tennis Court Road, Cambridge, CB2 1GA UK; 2 Institute of Biomedical Research, Shanxi University, 92 Wucheng Rd, Xiaodian Qu, Taiyuan, Shanxi 030000 China

**Keywords:** Signaling pathways, Metabolic reprogramming, Energy storage, Stress resistance, Chronological lifespan

## Abstract

Studies on replicative and chronological aging in *Saccharomyces cerevisiae* have greatly advanced our understanding of how longevity is regulated in all eukaryotes. Chronological lifespan (CLS) of yeast is defined as the age-dependent viability of non-dividing cell populations. A number of nutrient sensing and signal transduction pathways (mainly TOR and PKA) have been shown to regulate CLS, yet it is poorly understood how the starvation signals transduced via these pathways lead to CLS extension. Using reporters whose expressions are induced by glucose starvation, we have screened the majority of the ‘signaling’ mutants in the yeast genome and identified many genes that are necessary for stress response. Subsequent analyses of the ‘signaling’ mutants not only revealed novel regulators of CLS, such as the GSK-3 ortholog Mck1, but also demonstrated that starvation signals transmitted by SNF1/AMPK, PKC1 and those negatively regulated by TOR/PKA, including Rim15, Yak1 and Mck1 kinases, are integrated to enable metabolic reprogramming and the acquisition of stress resistance. Coordinated metabolic reprogramming ensures the accumulation of storage carbohydrates for quiescent cells to maintain viability. We provide new evidence that Yak1, Rim15 and Mck1 kinases cooperate to activate H_2_O_2_-scanvenging activities, thus limiting the levels of ROS in cells entering quiescence. These findings support the recent advances in higher organisms that the flexibility of metabolic reprogramming and the balance between energetics and stress resistance are the unifying principles of lifespan extension. Future work to reveal how the metabolic switch and stress response is coordinated will help delineate the molecular mechanisms of aging in yeast and shed novel insight into aging/anti-aging principles in higher organisms.

## Introduction

Two paradigms of aging studies in *S. cerevisiae*, i.e., chronological lifespan and replicative lifespan, have led to discovery of many pro-aging and anti-aging factors (TOR/SCH9, Ras/PKA, AMPK and SIR2) that are functionally conserved in higher organisms, including mammals (Burkewitz et al. 2014; Enns and Ladiges 2010; Johnson et al. 2013; Poulose and Raju 2015). While the replicative lifespan (RLS) measures the potential of mother cells to produce daughters in rich medium, the chronological lifespan (CLS) determines the mean and maximum survival of non-dividing cells in starving conditions. Two lines of evidence suggest that metabolic shift from fermentation to respiration and the activation of stress response are both important for CLS extension. Work from the labs of Shadel and Barrientos indicate that CLS extension, mediated by calorie restriction or reduced TOR signaling, requires mitochondrial respiration above a certain threshold (Bonawitz et al. 2007; Ocampo et al. 2012; Pan and Shadel 2009). Studies from the labs of Longo, De Virgilio and our own suggest that reduced TOR/PKA signaling extends CLS via the activation of stress response dependent on the Msn2/4 and Gis1 transcription factors, and the Rim15 and Yak1 kinases (Wanke et al. 2008; Wei et al. 2008; Zhang et al. 2013). Mitochondria are the major source of ATP, but also a major source of reactive oxygen species (ROS) whose accumulation is detrimental to lifespan extension (Breitenbach et al. 2012). Therefore, metabolic switch to respiration and the control of ROS levels have to be coordinated to ensure longevity.

**Fig. 1:**
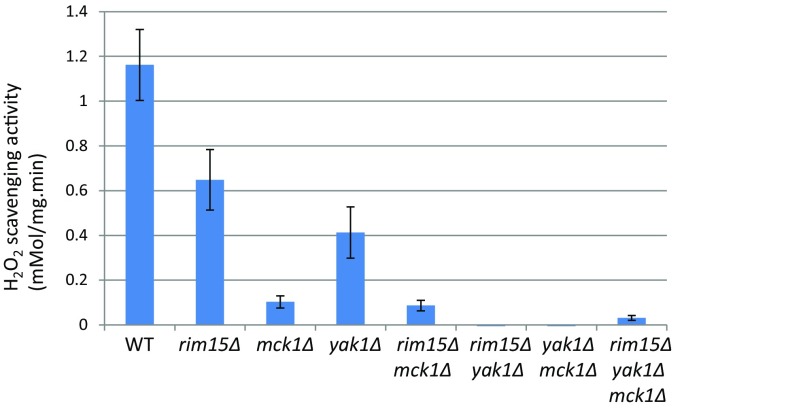
H_2_O_2_-scavenging activities in post-diauxic shift cells. Samples of WT and mutant cells grown in YPD were taken shortly after glucose is exhausted (12 h). Total protein was extracted by breaking cells with glass beads in Tris buffer (pH 7.5). The amount of H_2_O_2_ broken down (mM/min) was monitored at 240 nm and normalised to total amount of protein (mg) used in each assay to represent H_2_O_2_-scavenging activities. Mean value and standard deviation from quadruplicates were shown

**Fig. 2:**
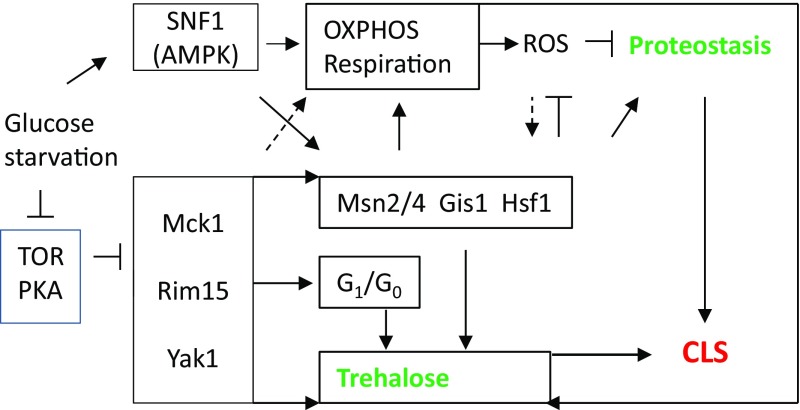
The current model of CLS regulation in yeast. Upon glucose starvation, a number of signaling complexes/proteins are activated (represented by *SNF1/AMPK*) or derepressed (represented by *Yak1*, *Rim15* and *Mck1*), which in turn promote mitochondrial respiration to accumulate storage carbohydrates (represented by *trehalose*). Yak1, Rim15 and Mck1 cooperate to retain transition-phase cells at G_1_/G_0_ by imposing a size threshold for S phase entry. The accumulation of storage carbohydrates, the antioxidant defense systems and the expression of molecular chaperones are transcriptionally activated by Msn2/4, Gis1 and Hsf1 transcription factors to maintain proteostasis

Based on this hypothesis, we screened the majority of mutants defective in ‘signaling’, using starvation-induced reporters (controlled by the *HSP12* and the *SSA3* promoters) whose expression is dependent on Msn2/4, Gis1 and Hsf1. Mck1, the yeast GSK-3 ortholog, was identified as a novel regulator of quiescence entry (Quan et al. 2015). Mck1 acts in parallel to the PAS kinase Rim15 to activate starvation-induced gene expression, the acquisition of stress resistance, the accumulation of storage carbohydrates (trehalose and glycogen), and the extension of CLS. Further genetic analyses revealed that the key factors for cell survival in stationary phase are the accumulation of sufficient storage carbohydrates (both trehalose and glycogen) and the elimination of ROS during the transition phases (Cao et al. 2016). The accumulation of trehalose and glycogen requires the integration of starvation signals transduced from multiple signaling pathways, including the energy-sensing complex (SNF1/AMPK), and the cell wall integrity (CWI) pathway, and the Yak1, Rim15 and Mck1 kinases which were previously shown to be negatively regulated by TOR and/or PKA. We have also demonstrated that the levels of intracellular reactive oxygen species (ROS) and the population size are controlled by Yak1, Rim15 and Mck1 kinases. Removal of any of the three kinase genes, especially *MCK1*, severely decreased the H_2_O_2_-scavenging activity in post-diauxic shift cells (Fig. 1). Removal of *YAK1* from the *rim15∆* or *mck1∆* mutants, abolished such activity, suggesting that Yak1 may act in parallel to Rim15 or Mck1 to eliminate intracellular ROS (Fig. 1). These data support the observation that metabolic reprogramming to increase energy storage and the activation of anti-oxidant defence systems are coordinated by a set of key signaling proteins to ensure long-term survival (Cao et al. 2016). Coordination of storage carbohydrate accumulation and the antioxidant defence systems is effected in part through transcriptional activation by Msn2/4, Gis1 and Hsf1 (Fig. 2). This set of factors are responsible for transcription activation of mitochondrial respiration, the antioxidant defence systems and the expression of molecular chaperones (exemplified by HSP and SSA proteins) (De Virgilio 2012; Morano et al. 2012). Based on these findings, we have proposed a framework for further studies to address the molecular mechanisms of quiescence entry (Miles and Breeden 2017), stress response (Ho and Gasch 2015) and chronological lifespan extension in yeast (Fig. 2). In this perspective, highlighted below are areas of research in yeast which we believe will further advance our understanding of aging principles in other eukaryotic organisms.

## The roles of Trehalose

The accumulation of storage carbohydrates, especially trehalose, is essential to CLS extension (Cao et al. 2016). Trehalose protects stationary-phase cells against various stresses (Eleutherio et al. 2015), supports cell cycle progression in poorer nutrient conditions (Ewald et al. 2016) and fuels quiescence exit upon return to growth (Shi et al. 2010). The performance of baker’s and brewer’s yeasts largely relies on their capacity to accumulate trehalose (Eleutherio et al. 2015). In plants, trehalose accumulation triggers autophagy during desiccation (Williams et al. 2015). Mammals do not synthesise this disaccharide. However, trehalose administration decreases the levels of toxic protein aggregates in animal models of neurodegenerative disorders by inducing autophagy (Emanuele 2014). Recently, it has been revealed that trehalose can be actively transported into mammalian cells, inhibiting glucose uptake and promoting autophagy via AMPK and ULK1 activation (DeBosch et al. 2016; Mayer et al. 2016). Further studies on the functions of trehalose in stress resistance, autophagy and lifespan extension and on how the accumulation of trehalose is regulated will be beneficial to both biotechnology and medicine (Eleutherio et al. 2015).

## Mitochondrial ROS as an adaptive signal to extend chronological lifespan

The free radical theory of aging (Harman 1956) has recently been challenged by a number of findings that mitochondrial ROS are not always harmful and can stimulate pro-longevity pathways in animals (Wang and Hekimi 2015). It is generally accepted that severe mitochondrial dysfunction accelerates aging, whereas mild mitochondrial stress can induce a wide-ranging cytoprotective changes (both metabolic and biochemical), resulting in longer lifespan (Sun et al. 2016; Yun and Finkel 2014). In yeast, loss of cytochrome C oxidase promotes ROS accumulation (albeit mainly from the ER) and severely reduces chronological lifespan (Leadsham et al. 2013). Indeed, when mitochondrial respiratory capacity is maintained above a threshold level (40%), yeast cells can accumulate sufficient nutrient stores to enable stress resistance and CLS extension (Ocampo et al. 2012). Two lines of evidence in yeast are consistent with mitochondrial ROS acting as an adaptive signal to extend CLS. First, yeast strains with reduced TOR signaling exhibited enhanced mitochondrial ROS (superoxide anions) during the growth phase, resulting in reduced levels of ROS in stationary-phase cells and elevated lifespan (Pan et al. 2011). This mtROS-activated hormesis and longevity extension involves the activation of stress response dependent on Msn2/4 and Gis1 (Pan et al. 2011), and the Rph1-dependent epigenetic silencing by triggering a non-canonical activation of the DNA damage response pathways (Schroeder et al. 2013). Second, inactivation of catalases increases chronological lifespan through enhanced levels of hydrogen peroxide, which activates superoxide dismutase to inhibit the accumulation of superoxide anions (Mesquita et al. 2010). Although it is not clear what type of ROS acts as the ultimate signal, these studies suggest that ROS generated by mitochondrial respiration may feed into the regulation network that coordinates metabolic reprogramming and the acquisition of stress resistance (Fig. 2). In this respect, *tor1∆* mutants accumulates less ROS at the stationary phase (Pan et al. 2011) but higher levels of storage carbohydrates (Cao et al. 2016; Hu et al. 2014) than wild-type cells. It would be interesting to determine whether enhanced energy storage or reduced ROS levels play a dominant role in CLS extension in *tor1∆* cells. Similarly, it is interesting to investigate whether strains defective in catalase activities also accumulate higher levels of energy stores to support extended lifespan. Finally, future work should also include the reconstruction of the genome-wide regulation network and reveal how ROS signals are sensed and transduced to the components of this network (Fig. 2).

## Yeast CLS as a model to delineate basic principles of aging

Studies in different organisms, including mammals, have identified nine hallmarks of aging: genomic instability, telomere attrition, epigenetic alterations, loss of proteostasis, deregulated nutrient sensing, mitochondrial dysfunction, cellular senescence, stem cell exhaustion, and altered intercellular communication (Lopez-Otin et al. 2013). Each of these hallmarks is connected to undesirable metabolic alterations and all the interventions designed to delay aging, including calorie restriction, are thought to operate in the context of metabolic reprogramming to ensure efficient nutrient utilization and to enhance stress resistance (Lopez-Otin et al. 2016). Our findings that CLS extension in yeast is regulated by a signaling network coordinating metabolic reprogramming and stress response suggest that yeast CLS model shares the basic principles with those operating in higher organisms. Under normal laboratory conditions, yeast cells change their metabolism from fermentation (rapid growth and reproduction on glucose), to respiration (slow growth and reproduction on non-fermentable carbon sources), and to maintenance on storage carbohydrates and other recycled nutrients. Enhancing the capability of yeast cells to switch to respiration by either genetic (*tor1∆*) or environmental (calorie restriction) modulations further extends CLS. Conversely, severely compromising peroxisomal function, mitochondrial respiration, gluconeogenesis, or deleting the effectors of the quiescence program shortens CLS (Bonawitz et al. 2006; Cao et al. 2016; Garay et al. 2014; Kawalek et al. 2013; Leadsham et al. 2013; Ocampo et al. 2012; Wei et al. 2008). In mammals, normal energy metabolism is periodically shifted between glucose and fat oxidation by the mitochondrial machinery, in response to physiological and nutritional circumstances. Dietary restriction and other lifespan-extending measures regulates mitochondrial function and triggers metabolic switches from anabolism to non-toxic catabolism, thought to be coordinated by the activation of sirtuin and AMPK, and the inhibition of mTOR and IGF-insulin sensing pathways (Finkel 2015; Lopez-Otin et al. 2016). Therefore, CLS extension in yeast also involves similar principles of regulation to those controlling lifespan in mammals. Revealing how SNF1/AMPK, CWI (PKC1), and other effectors of the quiescence program (Yak1, Rim15, and Mck1 in Fig. 2) coordinate metabolic reprogramming and stress response via the regulation of mitochondrial respiration will provide an in-depth understanding of the molecular mechanisms underlying this metabolic switch in yeast, allowing the identification of evolutionarily conserved signaling and metabolic modules that are essential to metabolic flexibility and lifespan extension.

In summary, our and others’ findings support that the key to extend lifespan in yeast and other eukaryotic organisms may lie with their ability (and the opportunity) to metabolically switch to alternative fuels and, at the same time, to overcome the redox stress to maintain homeostasis. Metabolic disorders contribute to many age-related diseases, such as diabetes and cancer. Revealing how the signal transduction cascades, ROS signals and metabolic circuits are rewired during the shift to mitochondrial respiration in yeast will provide valuable insights into ageing mechanisms and age-related diseases in mammals.
